# Introducing ‘*Open Questions*’ article type

**DOI:** 10.1098/rsob.200074

**Published:** 2020-04-01

**Authors:** Martha S. Cyert

**Affiliations:** Department of Biology, Stanford University, Stanford, CA, USA

## 1. Introduction

*Open Biology* is expanding its scope to introduce a new article type, ‘Open Questions’, which highlights current developments in an area of basic biomedical science that is developing quickly and ripe for discovery. The articles are to be written for an educated lay audience, with the aim of communicating scientific advances to the public—especially potential private funders.

## 2. The need for *Open Questions*

In the current funding climate with decreasing government support for science, funding of basic research by private donors, charities and foundations is of increasing importance.

Approximately 40% of health research in high-income countries is funded by public and philanthropic funding organizations [1]. These organizations play a central role in the development of new knowledge and treatments, particularly in areas that are not sufficiently profitable [2]. For example, the involvement of public and philanthropic funding organizations has been key in the development of therapeutics to combat neglected diseases [2,3].

However, most philanthropists are not scientists and must be educated to understand the potential impact of funding developing areas of basic biomedical science. We believe that research scientists should address this challenge.

Several scientific groups such as *The Science Philanthropy Alliance* (SPA; a group of private foundations, donors and a few select university research partners), are dedicated to increasing philanthropic support for basic research. SPA works directly with donors to shepherd them through the sometimes daunting process of establishing a new funding mechanism. An example of an *Open Questions* format are the perspective pieces ‘Extraordinary Opportunities to Support Basic Science Research’ which SPA invites scientists to write to highlight potential funding areas and these are shared with their clients [4].

Another example is this perspective piece, ‘A path toward understanding neurodegeneration’ [5] that was the result of an initiative sponsored by the Kavli Foundation in 2015 where scientists from outside the field collected their perspectives on research into neurodegenerative disease. This highlighted the importance of funding cell biology research to yield insights into the basic cellular processes that are perturbed by disease to result in neuronal death.

We believe that *Open Biology* should take a leading role in creating a forum to publicize emerging, understudied and/or underfunded areas of biomedical research. The first *Open Questions* article will be written by geneticist and radiation biologist Tin Tin Su on non-apoptotic roles of apoptotic proteins**.** The article will highlight exciting new findings and future directions in the field.

## 3. How to submit an article

As the Royal Society's journal dedicated to biology at the cellular and molecular level, *Open Biology* considers a wide range of research topics in cell and developmental biology, molecular and structural biology, biochemistry, neuroscience, immunology, microbiology and genetics (see scope https://royalsocietypublishing.org/rsob/about#question1).

Authors interested in submitting an *Open Questions* article will need to follow the Royal Society Publishing instructions for authors when preparing articles for submission https://royalsociety.org/journals/authors/author-guidelines/. All submissions will be handled by *Open Biology* Associate Editor Prof. Martha Cyert. The criteria for assessment will include: timeliness and importance of the topic, potential impact, and novelty of the understudied and/or underfunded research area. The articles are to be written for an educated lay audience, with the aim of communicating scientific advances to the public, especially potential funders. They should be succinct, non-exhaustive review-type articles (*ca* 2500 words). Articles will be subject to the standard peer review.

## Data accessibility

This article has no additional data.

## References

[1] Viergever RF, Hendriks TCC 2016 The 10 largest public and philanthropic funders of health research in the world: what they fund and how they distribute their funds. Health Res. Policy Sys. 14, 12 (10.1186/s12961-015-0074-z) PMC4759950 26892771

[2] Røttingen J-A et al. 2013 Mapping available health R&D data: what's there, what's missing and what role for a Global Observatory. Lancet 382, 1286–1307. (10.1016/S0140-6736(13)61046-6) 23697824

[3] Viergever RF 2013 The mismatch between the health research and development (R&D) that is needed and the R&D that is undertaken: an overview of the problem, the causes, and solutions. Glob. Health Action. 6, 22450 (10.3402/gha.v6i0.22450) 24119660 PMC3796018

[4] The Science Philanthropy Alliance. *xo-files*. https://sciencephilanthropyalliance.org/what-we-do/resources/xo-files/ (accessed 25 March 2020).

[5] Kosik KS, Sejnowski TJ, Raichle ME, Ciechanover A, Baltimore D 2016 A path toward understanding neurodegeneration. Science 353, 872–873. (10.1126/science.aai7622) 27563087 PMC6028188

